# Pseudomentalization as a Challenge for Therapists of Group Psychotherapy With Drug Addicted Patients

**DOI:** 10.3389/fpsyg.2021.684723

**Published:** 2022-01-04

**Authors:** Giovanna Esposito, Silvia Formentin, Cristina Marogna, Vito Sava, Raffaella Passeggia, Sigmund W. Karterud

**Affiliations:** ^1^Department of Humanities, University of Naples Federico II, Naples, Italy; ^2^Azienda ULSS 6 Euganea, Padua, Italy; ^3^Department of Philosophy, Sociology, Education and Applied Psychology, University of Padua, Padua, Italy; ^4^Department of Humanities, University of Naples Federico II, Naples, Italy; ^5^Norwegian Institute for Mentalizing (IM), Bergen, Norway

**Keywords:** pretend mode/pseudomentalization, group therapy, drug addicted patients, micro-analysis, treatment integrity

## Abstract

One of the main challenges in group therapy with drug-addicted patients is collective pseudomentalization, i.e., a group discourse consisting of words and clichés that are decoupled from any inner emotional life and are poorly related to external reality. In this study, we aimed to explore the phenomenology of pseudomentalization and how it was addressed by the therapist in an outpatient group for drug-addicted patients. The group was composed of seven members, and the transcripts of eight audio-recorded sessions (one per month) were rated and studied. The interventions of the therapist were measured with the mentalization-based group therapy (MBT-G) adherence and quality scale by independent raters. Two sessions, one with the highest and one with the lowest adherence, were selected, and the clinical sequences of pseudomentalization were analyzed in a comparative way. The findings revealed that pseudomentalization does occur as a collective phenomenon, akin to “basic assumptions” of Wilfred Bion, which we reconceptualized in this study. Any pseudomentalization seemed to be reinforced by the therapist when she was presenting frequent and long interventions, when abstaining from the management of group boundaries, when providing questions focused more on content than on the mental states of the group members, and when not focusing on emotions. However, the ultimate source of collective pseudomentalization seemed to be the fear of the group members of being overwhelmed by painful emotions, mental confusion, and a loss of identity. The findings also indicated that the principles of MBT-G may be a good antidote to pseudomentalization.

## Introduction

Pretend mode is one of the pre-mentalizing modes of thinking that appears in the early years of the road of a child to “full” explicit mentalizing ability (Fonagy et al., 2002) and which can reoccur as a problematic non-mentalizing mode of thinking in adults, especially in individuals with a personality disorder. In this latter case, it is also referred to as “pseudomentalization” (Bateman and Fonagy, 2010). PM is characterized by apparent awareness of mental states albeit revealing the absence of some of the essential features of mature mentalization. Indeed, it presents itself as an excessive consideration of how other people think or feel without there being any authentic interest in the other (Karterud, 2015a,b; Bateman and Fonagy, 2019). Thoughts are separated from mental reality, i.e., they lack a personal-emotional grounding in lived experience, and the narratives tend to be ruminative and overly detailed. Thus, PM can also be seen as akin to intellectualization and rationalization (Bateman and Fonagy, 2019). Patients can discuss opinions about themselves, others, and the world in a discourse filled with words with a seemingly psychological content but being devoid of any deeper emotional meaning, e.g., presenting as psychological and quasiphilosophical clichés or “empty words.” This is the phenomenon that has been labeled pseudomentalization (Bateman and Fonagy, 2010).

At least three pseudomentalizing subtypes may be observed in clinical practice: (a) intrusive mentalizing, which is characterized by a certainty about mental states and a lack of any connection between thoughts and feelings; (b) overactive-inaccurate mentalizing, which consists of a preoccupation with mental states but featuring inappropriate interpretations and very little genuine curiousness about mental states; and (c) bizarre mentalizing, which refers to highly inaccurate mental state attributions and psychologically implausible mental state inferences. Globally, these three subtypes have in common a lack of any recognition of opaqueness and the developmental nature of mental states and an absence of any sociocultural contextualization of experience by reference to physical reality (Bateman and Fonagy, 2010).

Pseudomentalization poses a series of challenges for the psychotherapist. These have been described in various textbooks (e.g., Bateman and Fonagy, 2016), but empirical literature on this topic is rather scarce. In particular, in group treatment, we can frequently observe pseudomentalization, and this is a phenomenon, which it is simply impossible for group therapists. They face complex challenges since it may seem that the group members are involved in a productive reasoning, while, actually, they are avoiding an authentic mentalizing discourse. In fact, pseudomentalizing is often adopted in order to counter the emergence of strong emotions, particularly the primary emotion of fear (anxiety).

Pseudomentalization may play a temporarily defensive role in such groups. However, there is also a risk that it may become embedded in the group culture and hamper a healing mentalizing process (Fonagy et al., 2017). The emergence of strong affective content could foster a defensive stance in which the members remain focused only on the surface and neglect the possibility that the group and the other members could serve as sources of growth and change (Sierra Hernandez et al., 2015). Such a failure in the integration of affects into experiences is also highlighted in other theoretical models. For example, Multiple Code Theory (Bucci and Maskit, 2007) is a general theory of emotional information processing that highlights the fact that the referential process makes it possible to communicate the emotional experience of a person to other people and to regulate emotions through the own words of a person or words of other people. When the referential process, which links sub-symbolic experiences with images and words, is not activated in the therapeutic relationship, an expansion of the emotional aspects can occur, which can hinder a positive outcome of the intervention (Mariani and De Coro, 2013; Esposito et al., 2019).

In this study, we aimed, first, to describe the phenomenology of pseudomentalization in group therapy, in this case the so-called Moviola group therapy approach, targeted at drug-addicted patients and, secondly, to investigate the role of the therapist in its appearance and disappearance.

### The Moviola Group for Drug-Addicted Patients

Several studies have demonstrated the close relationship between drug addiction and personality disorders, especially in the borderline range (Bannon et al., 2015). Accordingly, clinicians have to adopt intervention methods, which are not exclusively targeted at symptom recovery but rather at the development of psychic functions, such as mentalization (Esposito et al., 2020a), which tend to be highly compromised in the case of substance abuse. The impairment of mentalizing abilities in drug-addicted patients is also demonstrated in neuroscience (Gabbard et al., 2006), and developmental psychology has highlighted how drug addiction is related to attachment disturbances (Flores, 2004). Although there are not many empirical studies on this topic, mentalization-based treatment (MBT) for the drug-addicted patients has provided encouraging results, both in terms of improving personality functioning and of decreasing substance use (Morken et al., 2017).

Sudden failures in mentalization before and after relapse in drug and/or alcohol use can be observed in drug-addicted patients. Low levels of mentalization are also related to the habit of controlling one’s own mental processes through manipulating one’s own neurotransmitters by chemical means, e.g., through external, and not intersubjective, means. Often, people who habitually use substances lose their ability to recognize and reflect upon their own mental states and come to live in a mental and emotional reality that becomes more and more fictitious, strongly marked by the effect of the substance (Correale et al., 2014). These mechanisms cause an individual mistakenly to recognize herself/himself and to feel “real” when she/he is under the effect of the substance, actually when she/he is in a state of deviant emotional and behavioral activation. In this way, her/his original personality matrix becomes hidden by a false identity in which the substance fulfills the desire to escape from the frustrating company of oneself, to exit from the depths of the mind of a person, and to find a more satisfying existence in the fictitious bliss of drug-induced feelings (Correale et al., 2014).

From this premise, work on mentalization with drug-addicted patients has a fundamental value. In particular, the recognition of the opacity of mental states can be a starting point from which clients who are addicts may understand the value of exploring the mind instead of making judgments about behavior. Undertaking an activity based on mentalization means, first of all, gradually coming into contact with emotions that confuse the “thinking mind.”

These premises are the basis of the Moviola Group observed in this study. This was originally a therapeutic group targeted at cocaine users, meeting in an outpatient service at a department for addictions in a city in northern Italy. At a later stage, in order to respond to new forms and types of addiction, it was decided to change the target population and the working focus of the group, taking into account two characteristics: polysubstance abuse and a younger age. For this reason, the original Cocaine Group became the Moviola Group, addressed to young adults (from 20 to 30 years old) with polysubstance abuse (a mixture of alcohol and/or drug abuse, e.g., cocaine, heroin, or cannabis), criteria which characterize an increasingly numerous range of users in modern societies. The change in the name of the group from the Cocaine Group to the Moviola Group was intended to shift the focus from the symptoms to the process. The objective of the Moviola Group is focused on *thinking together*, in the here and now, through the “slow motion” of events and situations, as narrated by the members of the group. Indeed. the term slow motion (in Italian “moviola”) refers to a movement that is recreated in the narration of events in an attempt to offer the possibility of collecting the different points of view of the group members about what, in their opinion, the protagonists of the events narrated have in mind. The primary task of the group is, therefore, to recount the events, allowing different interpretations and taking into account the various points of view and experiences reported by the group members, while, at the same time, listening to the emotions of the protagonist in order to ground their own experiences. The activation of this “slow motion” process also allows the therapist to work on the interactions between the members of the group during the session and to use what happens in the here and now as an object of mentalization. The idea of slow motion as a group task was inspired by the MBT group therapy model (Karterud, 2015a), which we will now briefly describe.

### Mentalization-Based Group Therapy

Group therapy has from the beginning been an integral part of MBT for personality disorders, in particular for those in the borderline spectrum (Bateman and Fonagy, 2016). The principles for the group component have been spelled out by Karterud (2015a). The primary task is to promote the understanding of the group members of their own and others’ mental states, both in the context of the here and now and in the narrative context of interpersonal events narrated by patients in the group. By focusing on the emotions involved and on their attachment implications, the treatment as a whole aims at personality integration and development.

The realization of this task depends heavily on the leadership abilities of the therapist, e.g., in constructing a group structure and culture that serve as a fertile ground for the development of mentalization and affect integration and interpersonal trust (Karterud, 2015b). A group that more specifically favors experiences of a “safe base,” developed by means of firm leadership, and that improves the communication of affective and mental states can constitute an important maturational ground for overcoming resistances and enhancing the reflective capacity (Black, 2019; Esposito et al., 2020b). Moreover, research on the treatment of personality disorders has shown how poorly structured interventions, favoring the emergence of unconscious content and the overcoming of repression, are particularly difficult for borderline personalities due to the deficient structuring of their inner world, e.g., presenting as vague boundaries and polarizations in their self and in other representations (Levine, 2017).

Starting from these assumptions, Mentalization-Based Group Therapy (MBT-G) advocates a therapist style that adopts an active attitude in regulating the process of the session and, at the same time, respects the principle of not-knowing (Bion, 1963) in the approach with the patients (Indrehaug and Karterud, 2015). The therapist should try to balance these different tasks, e.g., being an expert in the management of the group, in maintaining a managerial attitude in regulating the phases and in ensuring the participation of all, but, at the same time, respecting the principle of opacity of mental states when intervening and deciding when to expose their own mental states for therapeutic purposes. Overall, the therapist should encourage the patients to maintain active and exploratory attitudes and counteract passive and dependent positions in the therapeutic process.

The therapist is specifically involved in the following tasks: (1) structuring the group, (2) exploring events, (3) involving group members in the work of exploration, and (4) regulating the emotional “temperature” in the group (Karterud, 2015a). These tasks require an active, alert, and authoritative management, both toward the individual members and toward the group as a whole, while simultaneously maintaining a position of curiosity and openness to mental states.

The combination of an active authoritative management and a compliance with the not-knowing stance are two of the key elements of an MBT-G group that distinguishes this model of intervention from the classic group-analytic approach. On one hand, both models take into account the dynamics of the group and the existence of an unconscious communicational group matrix that affects the relationships between the patients, the patients and the therapist, and the group as a whole; on the other, the models differ in the conceptualization of the role of the therapist in constructing this group matrix and in facilitating the therapeutic processes (Karterud et al., 2019).

Ideally, the culture of an MBT group becomes increasingly a field of possibilities, of asking oneself and others how one feels at a given moment or in a given situation, of wondering what thoughts and emotions are present in one’s own and in minds of others, and of allowing the patients to realize that they have thoughts and emotions that can be recognized and shared. In this field of possibilities, the patients can perceive a lesser sense of emptiness and deconstruction, acquiring greater intelligibility of their own mental states and those of others. The comparison with others is facilitated by the occurrence of group events that offer the possibility of understanding and communicating the mental states of a person, allowing a work of legitimizing the emotions of a person through mirroring (Pines, 1984), a process through which it is possible to see and recognize oneself through the reactions of others that are validated by the therapist and group members.

### The Moviola Group Seen From the Perspective of Mentalization-Based Group Therapy

One advantage of the MBT-G approach is that it is linked to a manual (Karterud, 2015a), which includes a rating scale of adherence and competence (the MBT-G-AQS). By means of this scale, it is possible to (1) rate the interventions of the therapist for specific group sessions and thereby identify, by a scientific method, group sessions that demonstrate high, as opposed to a low, adherence and quality, and (2) assess the individual interventions by the therapist in a micro-analytic study (Karterud, 2018). Although the Moviola group observed in this study is not conducted in strict accordance with MBT-G guidelines, it is inspired by that approach, and, therefore, it seems meaningful to study its processes through an MBT-G lens. Besides, the object of our study, pseudomentalization, or PM, is a phenomenon that occurs in all groups. However, there is no other method that captures the essence of this occurrence more effectively than the MBT-G-AQS. By applying this method, we can detect phenomena and their causal connections on both micro- and macro-levels.

### Objectives

In the present study, we aim to explore the challenges that drug-addicted patients pose to the therapist from a mentalizing perspective, with a specific focus on PM, and to examine the strategies adopted by the therapist in order to handle it. Specifically, our research questions are: (a) How does PM appear phenomenologically in sessions of the Moviola group? (b) Are there any differences with respect to PM between sessions, which are conducted with a high, as opposed to a low, level of integrity with respect to the MBT-G model? and (c) What is the role of the therapist in relation to the PM phenomenon and which kind of interventions seems to prevent or, alternatively, promotes, PM sequences?

## Materials and Methods

### Participants

Seven patients who had attended a motivational psychological path for at least 6 months at the outpatient clinic of the addiction department were recruited: six men and one woman with an average age of 24 years. All had a diagnosis of substance addiction, particularly to cocaine and cannabis, while some had a diagnosis of alcohol addiction. Almost all had experienced a period of at least 1 month of abstention from drugs, although some continued to have periodic relapses.

The group therapy was held from January 2019 to July 2019, for a total of 28 sessions. The group was conducted by a psychotherapist with the presence, mostly silent, of a nurse.

The participants signed informed consent in accordance with the ethical principles of the Italian Association of Psychology. This informed consent allowed the collection of narrative materials and audio recordings of the sessions to be used for training and research purposes. All the data were collected in accordance with the Code of Ethics of the World Medical Association (Declaration of Helsinki) and the Italian Law on Privacy and Data Protection 196/2003.

### Therapist

The therapist was trained in group-analytic psychotherapy and had worked with patients suffering from addiction problems for 13 years. She did not have any specific training in MBT-G, but she was inspired by this model when she decided to give a new structure to the group therapy for patients who are addicts, the Moviola approach.

### Methods

All 28 sessions of this group were audio-recorded and transcribed *verbatim*. Eight sessions (one per month) were selected and translated into English in order to be rated independently by one English and two Italian raters according to the MBT-G-AQS (Karterud, 2015a). After the sessions had been coded, a consensus rating was reached through discussion in the case of any disagreement and, given that one of the raters was an English-speaking coder, on the same occasion, any misunderstandings or ambiguities given by the translation were resolved. Afterward, the session with the highest treatment integrity with respect to the MBT-G model (Session 16) and the one with the lowest integrity (Session 12) were selected for further study since these sessions exemplified, respectively, good and poor handling of the pseudomentalizing sequences. Treatment integrity profiles for both these sessions were also determined. Next, in each session, we tracked the clinical sequences of pseudomentalization in order to analyze in a comparative way its phenomenology and how the therapist handled each sequence.

The MBT-G-AQS (Karterud, 2015a) was constructed in order to rate group therapist interventions in accordance with 19 items (see Table 1). The psychometric qualities of the MBT-G AQS have been thoroughly tested and found to be very good to excellent (Folmo et al., 2017). The first nine items are specific for the group setting and aim at evaluating the interventions with the therapist with respect to, for example, boundaries, group phases, turn-taking, exploring events, and engaging the group members in such explorations. The next 10 items refer to general MBT principles and concern, for example, interventions that promote a mentalizing stance and focus on emotions, non-mentalizing modes (including pseudomentalization), and patient-therapist relationships. All the interventions of the therapist are rated for adherence and quality. Adherence is a quantitative measure that reveals how many of the interventions of the therapist fulfil the requirements of the different items. It may range from 0 to 100%. The following items are not rated for adherence since, generally, they cannot be deduced by specific interventions but are conveyed by more general attitudes: Item 6 (care for the group), Item 7 (managing authority), Item 10 (engagement, curiosity, and warmth), Item 13 (regulating emotional arousal), and Item 15 (handling pseudomentalization). Interventions that receive an adherence rating may also be rated for quality. However, for practical purposes, the quality ratings are assessed for the total session. Quality refers to the level of skill in intervention delivery by the therapist and is rated on a Likert scale from 0 to 7, divided into four levels: (a) not applicable (0), which is assigned when the intervention is not observed and not judged as essential; (b) low (1–3), which is assigned when the intervention is delivered with a poor quality or when relevant events in the group occurred and the therapist did not comment upon them; adequate (4), which is assigned when the intervention is delivered in a “good enough” manner; and high (5–7), when the intervention is delivered with a very good or excellent quality.

**TABLE 1:** Definitions and examples of MBT-G-AQS (adapted from Karterud, 2015a,b).

Items	Definition	Examples
**Group specific items**		
1. Managing group boundaries	Management of boundary-relevant events (such as absences, new members, delay)	T: You were absent last time, C. We wonder why.
2. Regulating group phases	Active role in dynamic management of session structuring (opening, middle and closing phases)	T: Let us start with some reflections on last group meeting.
3. Initiating and fulfilling turn-taking	Facilitating mentalizing turn-taking	T: OK, let’s start with C. You want to explore something with us.
4. Engaging group members in mentalizing external events	Engagement of group members in exploration of events brought up in the group	T: What do you all think about the story C told us?
5. Identifying and mentalizing events in the group	Identification of relevant events in the group and mentalize them	T: Seems like you, patient A, reacts to something here …
6. Caring for the group and each members	Making the group a secure base for the members	T: Unfortunately, I will be absent next time, but my colleague B, which you know, will conduct the group
7. Managing authority	Maintaining an authoritative role in leading the group	T: I know this is painful, but we cannot avoid dealing with it in the group
8. Stimulating discussion about group norms	Working on normative group-as-a-whole issues	T: Anger in groups may be difficult. How should we handle that?
9. Cooperation between co-therapists	Building a confident cooperative relationship between the co-therapists	T: I feel a bit confused. What do you think, Therapist 2?
**General items**		
10. Engagement, interest, and warmth	Attitude of authenticity, openness, engagement also through non-verbal signals	T: It makes me sorry to hear this, C. Hope you recover.
11. Exploration, curiosity, and not-knowing stance	Assisting group members in an exploratory process and stimulate this process	T: I am curious to know what other group members think about your reaction when your mom called you
12. Challenging unwarranted beliefs	Sensitive challenging of fixed, clichéd-like, unwarranted beliefs	T: What do you mean when you describe yourself as stupid?
13. Regulating emotional arousal	Maintaining of an ideal emotional arousal to foster mentalization	T: Just take your time, C. We can come back to this painful theme later.
14. Acknowledging good mentalization	Support and praise for members’ good mentalization	T: Seems like you handled this better this time. What do you think was different?
15. Handling pretend mode	Recognizing and handling sequences of pseudomentalization	T: I must admit I have a hard time concentrating. What are we exactly talking about?
16. Handling psychic equivalence	Contrasting and handling concreteness of thought	T: You say nobody in this group likes you. Let’s stop there and explore that.
17. Focus on emotions	Maintaining a focus on emotions and their mentalization	T: This was a hard blow for C. Do you feel it too and what is your thoughts about it?
18. Stop and rewind	Interruption of destructive sequences and engagement in their review to regain good level of mentalization	T: Can we stop, please? I think we need to slow down. What happened?
19. Focus on therapist-patient relationship	Mentalization of transference and countertransference	T: Seems that some of you didn’t like the way I terminated the session last time.

## Results

First, we briefly describe the integrity profiles of the two selected sessions, and, thereafter, we discuss in more detail some of the clinical sequences. Our main focus will be on the relationship between the clinical appearance of PM and the interventions of the therapist.

### Integrity Profiles of the Two Selected Sessions

#### Session 12

In Session 12, the total number of therapist interventions was 163. In these interventions, we found 50 occurrences that were rated as compliant with the MBT-G-AQS (31%). This percentage is a good indicator of MBT adherence (Folmo et al., 2017), and, in this case, it is low.

The overall quality of the session was also rated as low (Level 3). A higher level (5) of quality was achieved only for Items 6 (care for group members) and 10 (engagement, interest, and warmth), which may suggest a more supportive, than explorative, style of leadership. Notably, the quality of handling PM was rated at Level 2 (poor).

#### Session 16

In Session 16, the total number of interventions of the therapist was much lower, 43. Here, we found 41 occurrences that were rated as compliant with the MBT-G-AQS (98%), indicating much higher adherence to MBT principles. In other words, the therapist intervened less often, but, when she intervened, in the majority of cases, it was in accordance with the MBT guidelines.

The overall quality of Session 16 was also high (Level 6). None of the 19 items were assigned a quality rating below adequate (Level 4). The handling of PM was rated at Level 4.

### The Course of Session 12 With Special Emphasis on the PM Sequences

In what follows, we report *verbatim* transcripts of chosen clinical sequences. After each therapist intervention, we have indicated the number of the item of the MBT-G-AQS scale (A1, A2, etc., see Table 1) that is represented in the intervention according to the consensus of our raters. When no item is marked, it means that the intervention cannot be identified as a specific MBT-G intervention.

At this group session, 8 patients attended, although several of them were late. Patient A was missing, but he had been observed around the venue before the meeting. At the previous session, Patient A told the group that he was not able to remain abstinent, which was a requirement for group participation, and that he intended to approach a therapeutic community for more extensive help. His message stirred up diverse reactions. The therapist thought that it might have shattered the still vulnerable trust within the group.

Therapist (TP) starts the group by saying: *Well guys*…

Patient M: *But is anyone missing?*

TP*: No, I haven’t received any message! They may be a little late, but they will arrive. In the meantime, we will begin. Let’s start a little with you. How are you? I don’t know, I see some.*

Patient M: *A strained week for everyone.*

TP*: Spring never brings good things, right?*

Patient NU: *Yes.*

TP: *It is always a somewhat destabilizing period. At least, I don’t know, this period here is a bit difficult every year.*

Patient M*: Why do you say so? For what reason?*

TP: *Maybe the days get longer, maybe they affect people’s mood a little, it’s a bit of a period, it’s hotter, isn’t it? Temperature changes, in short, and some people are not able to take it in their stride but sometimes it affects those who are very sensitive to changes also - Hello R.! Did you see the others outside?* (**A1)**

Patient R: *Yes, there was MK and*

TP: *And why are they waiting to enter?* **(A1)**

Patient R: *MK was on the phone with his*…

TP: *Ah, with his wife, girlfriend*

Patient M: *MK should turn off his phone in my opinion*

Patient R: *I apologize for the delay, I arrived home late from university and then well*…

TP: S*omeone is ringing*…

Patient R: *Yes, yes, yes, yes, yes.*

TP: *Eh, sorry, but they rang, and I think it’s the others.*

Patient R: *Did I miss something?*

TP: *No, no, no, we have just started, we were warming up the engines a bit, as we usually say. We were taking a warm-up tour but, in reality, I was talking about spring! The fact that it’s always a bit turbulent* **(A2)**

Patient M: *For me, if it weren’t for the job*…

Patient NU: *Me too*

Patient M: *I like the days with more sun*

TP: *Yes, it is definitely positive, but those who have a bit of*… *usually long days can stimulate craving. In short, those who have certain problems are more sensitive here*

Patient NU: *Mostly season changes are always*…

TP: *Here, exactly, is the phase itself.*

What we see here, right from the beginning, is a classic PM sequence. Formally, the theme is about mental states (“how are you,” “people’s mood,” and “craving”), but what is supposed to affect these mental states is the weather and the season. We are about halfway through this sequence, and the topic of the absent member A pops up again. The group members air their frustration. However, at this point, the therapist tries to structure the session: “*No, besides A, surely we have many other problems here. Who*… *who do we want to start with?*”

No one particularly enters the scene and the topic of A surfaces again. Group Member F suggests that there is a group problem, e.g., that “*we don’t see the group as a refuge, we live it like a gallows.*” There are disagreements but some realization that it is difficult to “open up” and “tell one’s story.” At this point, the therapist enters with a long intervention, in fact composed of 450 words. It starts like this:

TP: *It seems to me you still are attached to this group. There is an affection*—*I feel it, I see it, in short. However, it is true that each group acts as a mirror in the sense that you see yourself just as you are, based on what others send you back. That is, you can also try to distract attention, emphasize only its positive aspects, but, for better or worse, then the others discover you, right? They tell you, look, I don’t see you well! So, maybe, maybe finding yourself in front of your mirror is not always so positive, is it? Seeing things as they are, seeing the problems I have that I don’t want to have, but that others see. It can sometimes be experienced as a gallows, can’t it?*

The problem with such long interventions, although the content may be “correct” in a way, is that the therapist risks talking above the heads of the participants; the argument becomes too complex and transcends their attention span. Frequently, in this session, the therapist turns to the group, asking: “*do you understand?*” taking an authoritative stance that tends to establish principles and rules in a top-down direction as well as directing the discourse to a determined pedagogical end. Usually, people do not like to appear foolish, so, if uncertain, they will often *pretend* that they understand. Besides, the therapist is a discourse model and the participants will tend to imitate her, for better but also for worse. In this case, Patient R responds with a very long comment (330 words), which contains sentences like this:

Patient R: … *Basically, the other thing is that shame is subjective. Up to now, even if I have said things that, thinking about it objectively, are not that beautiful, I have never, until now, tried, let’s say shame. In the sense that I have more than ever acquired an awareness, which is not a rational thing, that is, it comes from within.*

The content of the group discourse now moves from relationships to parents, particularly fathers, and what the therapist labels “the paternal function.” The problem with this discourse is that it is dominated by *opinions*, opinions about how (ideal) parents and children should behave and what might go wrong and initiate, in the worst case, a descent into drug addiction. It is a discourse with a PM flavor and, similarly to the opening phase sequence, mostly of the intrusive subtype, lacking deeper personal emotional experiences and decoupling a psychic from external reality.

TP: *And yes, of course, the teenager continually challenges the limits*

Patient M: *The teenager but also people. not just teenagers*

Patient F: *I did what I wanted.*

Patient M: *And therefore, it takes limits, rules*

Patient F: *Eh, A must therefore also grow up at this point here.*

TP: *But even for you, growing up means being able to put these limits on yourself, without obviously having the parent to put them there. It is a paternal function that you introject, it is said, that you learn to use with yourself. For example, you yourself set limits, but everyone has to set limits in life, to be able to work, to be able to go to work in the morning, like, I have to set myself a limit, it’s not that I can stay out until two in the evening at night if the next day I have to get up and come to work, do you understand? But I no longer need a parent who tells me, I learn. For living, this is a little the paternal function in a broad sense* **(A7)**

Patient M: *Being responsible for your actions*

TP: *Having limits in mind*

Patient N: *I can say that I also have limits, that is, I set limits and I achieve them, there. But if it was like two years ago, I had no limits*

Patient M: *They can be limits on money, friendships, work, schedules. They can be any kind of limits*

TP: *Exactly, yes*

Patient N: *Now I can also say, look, I do this, I don’t do this*

However, some personal experiences do surface, and the therapist addresses them, although in a rather “individual therapy in group” manner. In this atmosphere, Patient S for the first time talks about his family history. He has to be pushed a little before he starts; he would prefer to do it “next time.” It is a sad story about his Italian mother and African father, about the death of his mother, and his adoption by his grandmother. The story does not contain that much reflection, but it is personal and painful and indicates another type of discourse rather than PM.

After a new round of opinions about fathers, limits and drug use, Patient M talks about “*how deeply we have disappointed*…” our parents, and patient F responds: “*Me above all*… *I sold the gold*….” Therapist: “*Did you sell the house gold? How much? Ten thousand?*” Interestingly, the group members continued their discussion about fathers as if nothing had happened. It is as if the words of Patient F did not count, as if they were not (really) real, until the therapist invited Patient F to tell the group more, and he talked about this incident, now with the other group members listening and participating, about when he stole 10,000 euros of the family fortune and had fun for a week. Patient F added that “*He (his father) still loves me a little, but let’s say he hates me so much. It also annoys him to see me. I really see that on him. He looks at me like I’m shit*….” All of a sudden, the group was not pretending anymore. It was filled with painful feelings, above all feelings of guilt and confusing thoughts. However, this reality is hard to cope with and, when another group member made a similar confession, that he stole 2,000 euros from his parents, the group avoided exploring it.

After a while, the therapist turned to female Patient C, and somewhat reluctantly, she entered the scene. Patient C also had a sad family story to tell, and, most importantly, she found the courage to talk about how she was sexually abused after getting drunk at a disco. After that incident, her alcohol addiction started. Again, reality fell heavily upon the group members and, with the help of the therapist, they tried as hard as possible to understand and support Patient C in her narrative of the trauma, which, previously, only her mother and best friend had known about. Patient C described how in the past she had pretended that the incident had not happened. However, in the group, the pain was palpable.

### The Course of Session 16

Session 16 starts with an important premise. Group Member MK had been denied access to the previous session (15) because of an aggressive outburst in Session 14. Now, in Session 16, the therapist assumed a more authoritative leadership style, right from the beginning, in the opening phase:

TP: *Only M is missing because he is sick, but he greets you. I would also like to inform you that today A has entered the therapeutic community. Everything is going well, and we are hopeful. The last time MK was absent, he was a little under stress. Now he’s back, I hope you’re a little more relaxed. Then if you want to say something to the group about what happened we’ll start with a little from you. Then, if I am not mistaken, there will be a bit of time to give to C who has left us in suspense with respect to some of her decisions. And I have also observed that perhaps wants to talk about the very hard days that he has had in this period. Then I don’t know if even F, N and R want or need to say something.* **(A1, A2, and A3)**

To this introduction, Patient MK responds directly:

Patient MK: *First of all, I would like to apologize to the group and in particular to M (who is absent) and to R for the outburst I had last session before my absence. I don’t know exactly what you perceived, but surely it was my outburst. However, I didn’t want any of you to be offended, and I hope it wasn’t a bad example. This might illustrate that everyone can lose their patience. It used to happen to me much more often when I was using cocaine in the past. When I got angry, it was certainly not a pretty sight. I don’t know what you perceived. You can lose your patience, but you always have to keep calm and to stay focused. For me, recognizing that I’m wrong and apologizing is something new that I’ve never done before. After such moments of anger, you don’t even remember what you said. I had a meeting with the therapist, and I revisited things a little. I was under so much stress, and it’s not easy for me to hold off the fact that I do too many things. I always get upset when things don’t go according to my plans. Even in my daily life, I tend to react with anger and to make intimidating remarks. I still have to work a lot on this.*

It is useful to compare this opening of Session 16 to the opening of Session 12. There is not much pretending here. It is straightforward, honest, and highly relevant. The group then proceeded with a fine sequence that mentalized the event in the group when Patient MK lost his temper. Most of the members commented on how they perceived the event and reflected upon it with the help of rather short and direct interventions by the therapist:

TP: *But, of course, in a group you can also let off steam. That is not the problem in itself. What we reflected on together during the session that you missed is that there is a limit to what you can achieve.* **(A7, A17)**

Patient M: *I missed the situation by my own hand. I am not a superhero, it happened to me and I apologize. The important thing for me now is being able to apologize. I thought about it a lot during the days that I didn’t come to the group. The important thing is to improve. I still have work to do on patience. I’m not a quiet person, I can’t sit still, I still have to work on patience.*

TP: *And as you said before, also on the fact that you can’t pretend to have everything under control, don’t you? Does this thing stress you?* **(A17)**

Patient M: *I should take some space for myself, to unwind. Otherwise, I get too charged up with tensions. I should manage my day differently. I don’t always succeed. Here it is clear that I didn’t want to offend anyone, and I apologize to the group.*

Patient F: *M., I would like to tell you that in any case you have lost your patience on a difficult topic for you. You are facing your life well, but the subject was a sensitive topic for you.*

TP: *Yes F, you say something important for M., but now we are trying to evaluate the way he has managed his anger* **(A17, A4)**

Patient M: *I (turning to F) was not able to handle the anger. It was the way I took it that wasn’t good. It was a useless outburst against the institutions and against that guy.*

TP: *But maybe it’s how you managed it in the group, right? It is the anger that you have not managed in your relationship with the group, not so much in relation to the person you were talking about. I do not want to open up the subject of your contention with that person again, but we are talking about how you handled it here and perhaps how the group experienced your anger* **(A4, A17)**

Patient M: *At that moment, maybe I was looking for solidarity from the group, maybe I was trying to make them get as mad as me with that person and instead seeing that they didn’t agree with me and were even trying to make me think, my anger increased even more at that moment. I was looking for someone to tell me revenge you are doing right, you have to take revenge.*

TP: *So, the problem is the difficulty in accepting what others tell us against our expectations?* **(A16)**

Patient R: *Yes, It is not so much what you said, it is just how you answered M. You were agitated. While M and I told you things in a low tone, you answered in another, do you understand? I’m glad you’re apologizing, but this confrontation is useful to understand what happened at that moment.*

In this sequence, the therapist emerges as an expert in group dynamics rather than in the content that emerged in the discussion that led to explosion of anger of MK.

TP: *But what you call obvious may not be in the other’s point of view, which is sincere at that moment one commits to tell you that thing. To you it seems obvious but the point is to respect what the other has to say. It’s that sometimes we want to hear others say exactly what we expect. Isn’t it?* **(A12, A16)**

The therapist highlighted the importance of respecting different points of view as an element of group therapy and also invited silent members to talk about all these incidents. This was carried out without falling into generalizations but by remaining in the event that had happened and that everyone had experienced directly. The validation of the existence of different points of view in the group seemed to allow Patient MK to reach other points of view in his own mind, related to the observation of his own behavior in the group and the diverse reactions that the different members of the group presented with respect to his anger and the content he brought. There were those who supported him and those who wanted to express a different opinion, but he recognized that what he wanted was only that his own point of view was defended, and he connected this expectation with the mental state of anger and his unwillingness to manage it at that moment: “*In particular, R and M made me crazy because they didn’t support me. I was more pleased by the fact that F tried to support me, but I didn’t accept any points of view different from my own at that time.*”

After this important sequence with MK, the therapist offered space to Patient S to talk about how he had been in the last few days. He shared with the group that he had relapsed but did not feel guilty about it. Some group members immediately started to declare their opinions about the feelings of Patient S prior to the relapse. The therapist stopped the ongoing inquiry: “*Sorry, but, for a moment, let’s try to let him talk a little bit? I didn’t understand how he felt exactly and what he wants to tell us right now. It’s not clear to me.* **(A18 A11)**.” When the group continued to press him, she stopped again to rewind: “*Sorry, but I still don’t understand what’s on his mind. S, do you want to try making hypotheses about the thoughts you had before drinking?* **(A11 A18)**.” Fostering the mentalization process through “stop and rewind” (Item 18) prepares the ground for interventions by both the therapist and the participants aimed at exploration, curiosity, and not knowing. Here, we see how, unlike Session 12, the therapist did not engage in any “individual therapy in the group” but invited everyone to participate in an exploration of the underlying mental state that connected to shame and fear of Patient S that his condition as an adopted child would be highlighted by a social worker.

In fact, after some comments characterized by certainty about emotions of Patient S that pretended to depict precisely what Patient S felt or thought before his relapse, several members of the group now assisted the therapist, on her explicit invitation, in the exploration of disclosure of S, and some of them even commented on it, modeling the therapist. They stopped talking in the place of S and joined the therapist in a not-knowing and genuinely curious stance. Patient F, in fact, said: “*What did you think before drinking and drinking? Let’s do the ‘moviola’ on this.*” Later, Patient R intervened, checking his understanding of mental state of S, instead of stating it as a certainty: “*I’m sorry I didn’t understand, you’re afraid of not been seen any more as a family member if the social worker comes to talk about you with all of them. I got it right?*” In this atmosphere of curiosity and exploration elicited by the therapist, Patient S could mentalize with the group his painful and confusing thoughts about his identity and family background.

At the end of the session, Patient C, who left the room due to dizziness, rejoined the group and shared with the group members her decision to enter a residential therapeutic community. She, in fact, realized that she needed more help because she did not “*want to stay in this shit anymore*,” even though the perspective of the community is fearful, and she regretted not being able to maintain her commitment to the group. Nevertheless, the response of the group was aimed at containing her and supporting her decision.

## Discussion

The main findings from this study are the following:

1) Several sequences of collective PM/pseudomentalization could be identified and their phenomenology could be described.
2) PM sequences are not universal in groups. We found PM sequences in one of the group sessions studied but not in the other.
3) The group therapist seems to play a significant role in the dynamics of PM: we could identify therapeutic interventions that seemed to promote PM and others that seemed to prevent it.
4) In this case, MBT-G principles seemed to be an effective antidote to the proliferation of PM.

PM flourishes in ordinary life when we chat, play, engage in discussions, talk about all or nothing, and just get along without things being “that important.” Politicians are expert at this when they can give long speeches without saying anything essential, labeled “bullshit” by the philosopher Harry Frankfurt (2005). However, in group therapy, it becomes a problem since therapeutic groups are invested with an ideal requirement for the containment and exploration of painful mental states. Accordingly, there is the need to identify PM and to counteract it.

In this study, we have identified several PM sequences. These sequences were definitely of a collective nature, and we may rightly speak about group discourse modes. They are ways of talking together that seem to be experienced by the participants as a meaningful way of being together but lack the personal and emotional commitment that the primary task of the group demands. As such, PM sequences appear to the observer as having a detached, or “as-if” quality (Bateman and Fonagy, 2019), although the content of the discourse seems to be concerned with mental states. The opening phase of Session 12, which we have described in detail, is a good illustration. In this case, PM starts immediately, but when does it end? It definitely ends after approximately half an hour, when Patient S starts to tell his sad story. During this 30 min, PM was, more or less, always present. It is like a strong undercurrent that surfaces periodically. It might be compared to the term by Wilfred Bion (1961), basic assumptions, e.g., something that undermines the primary task activity of the group from “beneath,” more specifically the basic assumption of flight (Karterud, 1989). In our group, it appeared as the tendency of group members to provide solutions and banal explanations or to insist that “*you have to understand (think, do, try*…*) that*…,” “*you did that, so you accept this*,” etc. Sometimes, they stated what the feeling of the other was like, for example, “*you felt lonely and bored!*,” “*you felt a weight*,” “*you lost confidence.*” During these sequences, the group discourse was centered around rules or guidelines on how to behave and feel in the “right way,” as in intrusive pseudomentalization where the opaqueness of minds and connections with emotional experiences are lost.

Bion (1961) suggested that basic assumption phenomena surfaced when the group was afflicted by overwhelming anxieties and should be considered as a collective defense mechanism. Certainly, in this group, when the participants started to talk honestly, open, and emotionally, almost unbearable memories of death, loss, adoption, betrayal, violence, rape, and theft were revealed. We may hypothesize that approaching these memories activated emotions that were too intense for the members to regulate effectively, and that, in order to counter the emergence of these strong emotions, they fell back on the non-mentalizing mode of pseudomentalization.

The above reasoning may also be an explanation of why the therapist behaved so differently in these two sessions. During the first 30 min of Session 12, the therapist was definitely an integral part of the PM discourse. During this sequence, the name of Patient A repeatedly popped up, indicating that the group was certainly preoccupied with him. He signaled a withdrawal from the group but was observed in the surroundings. However, the theme was never discussed. Why? Does his withdrawal shake an initial idealization of the group? Does this also agitate the therapist who responds by acting out a countertransference of detachment, until she gradually assumes a more competent therapist role?

The course of Session 16 demonstrates that PM is a fluctuating phenomenon, even in this group with so much pain to bear. This fact highlights the significance of the therapist. We have already speculated that, during the first 30 min of Session 12, she was aroused by her own countertransference fear. But, more precisely, what did she do differently in the two sessions? In Session 16, we could observe that the therapist warded off very effectively any pseudomentalizing discourse and that strong emotions found their place in the group narrative. We noted that, overall, in Session 12, the therapist intervened very often and with long and sometimes complex interventions of a more pedagogical type. In contrast, in Session 16, the therapist intervened far less often, and the interventions were mostly short and simple.

There were also important differences with respect to content. In Session 12, the therapist tended to determine the content to stop and change the subject without waiting for the group. This attitude was reinforced by a frequent use of the expression “do you understand?” The therapist was caught up in formulating explanations and theories that the group members had to align with or not. The patients seemed to replicate in a way the attitude of the therapist toward the other group members by imitating the model of intervention of the therapist. Indeed, the members formulated explanations, instead of exploring the mental states of the others, and provided solutions and rules of behavior for different contexts. In Session 16, by contrast, the therapist seemed much more focused on the process than on the content, and her interventions tended to be more supported by curiosity and doubt than by the pursuit of rational explanations. The expression “do you understand?” never appears in this session.

PM also seemed to be stimulated by the therapist, using a language of complicated words and concepts, presumably going “above heads of the members.” That too seemed to be imitated by some members. A different therapeutic modeling occurred in Session 16, where the members were stimulated by the therapist to explore their own and others’ mental states to focus on emotions and to engage in mentalizing external events. During this session, indeed, the therapist was more directive in orienting the group discourse toward mentalizing aims, in structuring a kind of turn-taking and in frequently stopping and rewinding the group discourse when it seemed to lose sight of mentalizing objectives.

Since PM could not be observed to any substantial degree in Session 16 and since the way of the therapist of conducting this session was more strictly in accordance with MBT-G principles, can we conclude that there is a causal relationship between these phenomena? Not in any “hard” sense. However, we will argue that these phenomena, to a significant extent, are related. After all, we have to emphasize that the principles of MBT-G were constructed in order to counteract PM and similar collective regressions. Thus, we will take the liberty of postulating an inverse relationship between PM and MBT-G treatment integrity, specifically to mention the most important interventions, when the therapist creates bridges with previous sessions, manages group boundaries, structures the group, and engages the members in mentalizing current and past events while containing and focusing on current emotions.

The good news in this story is that groups with poorly functioning patients are not doomed to remain in unproductive or destructive group discourses. When Bion (1961) formulated his basic assumptions theory, it came with a rather pessimistic therapeutic message. He could not foresee any therapeutic style that would “rescue” therapeutic groups from basic assumptions functioning. However, the way of reasoning of Bion had significant limitations. He was stuck in drive theory and the theory of Melanie Klein of early psychotic anxieties. Moreover, his phenomenology was flawed. He postulated a basic assumption of “fight/flight,” supposing that “fight/flight” was a unifying concept. However, Karterud (1989) was able to demonstrate that fight and flight were different emotionalities in groups; they did not always come in one package. A modern reconceptualization of Bion’s original idea is that group rationality (or mentalizing capacity) may be undermined by (contagious) primary emotions and that FEAR (as described by Panksepp, 1998) corresponds to the basic assumption of flight. Actually, what we have found in this study, as conceptualized by the more modern concepts of PM and pseudomentalization, corresponds quite well to the basic assumption of flight as identified by Karterud (1989). Flight is driven by the primary emotion of fear. So, what are the people in this group afraid of?

In the introduction, we discussed the need for individuals who are drug addicts to “distort reality” and defend the “fictitious reality” constructed by substances in the mediation of the relationship between the mind of the patient and her/his environment. When we studied this group carefully, we came across, in no more than two sessions, painful memories of death, loss, adoption, betrayal, violence, rape, and theft. The fear of being overwhelmed by these memories, with their inherent emotions complicated as they are with secondary feelings of shame and guilt, e.g., not being able to approach them and mentalize them, seems to us to be the ultimate source of PM as a defensive move. The sad destiny of many individuals who are drug addicts is not only painful traumas from early childhood but also traumas and humiliations extending into adult life under the control of an addiction lifestyle. In this study, we have found several examples of an intrusive pseudomentalizing discourse, and we may hypothesize that addiction disorders, in particular, might be victims of this subtype of non-mentalizing thinking, characterized by certainty about mental states and a disconnection between emotional experience and social cognition. This decoupling may serve as a protective/defensive factor to counter the fear of emotional turmoil, confusion, and loss of identity. Furthermore, substance abuse might allow patients who are addicts to “feel in control” of their emotional states by shutting them down when they approach consciousness. In other words, we may say that, in some way, intrusive PM imitates the effect of such substances. Moreover, a certainty about mental states may be comforting and soothing. In fact, it is less threatening to be sure of what other people are thinking than wondering what is going on inside their heads. In the same way, it is easier to chemically turn off thinking and feelings than to face and mentalize negative emotions.

## Limitations

This study has several limitations. We identified a limited number of PM sequences and only from a group of drug addicts. A larger number of group sessions from diverse groups might have enriched the phenomenology and revealed more nuanced relations between therapist interventions and group processes. The study indicated a connection between the occurrence of PM and the behavior of the therapist, e.g., that certain interventions seemed to promote, and other interventions seemed to prevent PM, and that these therapist qualities could be captured by the MBT-G-AQS. Although this is in accordance with clinical literature, such a connection should be replicated. Furthermore, although we believe in the strength of the qualitative and phenomenological nature of this work, we also recognize that it could benefit from the matching of qualitative results with quantitative data with regard to the group process (such as therapeutic factors or cohesion). Moreover, it would be interesting in the future to collect and merge data from reports of therapists with evaluations of raters. This issue also has implications in terms of outcomes. As a non-mentalizing mode, pseudomentalization is, by definition, a sign of low reflective functioning. It might be expected that patients and groups, toward the end of the treatment, would display lower levels of PM than at the beginning of the treatment. However, this has not been verified empirically.

## Conclusion

From the study of this group of drug-addicted patients, we have verified that non-mentalizing modes of “pretending” do occur as collective phenomena, and that they are characterized as a kind of preoccupation with mental states that favors unwarranted causal claims and explanations (e.g., “seasonal qualities influence moods”) rather than genuine explorations of mental states. Furthermore, the study indicates that the group therapist has a strong influence on the occurrence of PM, although the ultimate source is, probably, the fear of the participants of strong emotions, mental confusion, and loss of identity. The PM seemed to be reinforced by poor boundary regulation, frequent and long interventions, and interventions with obscure content. It was probably also influenced by countertransference. On the other hand, PM seemed to be counteracted by a therapist style that adhered more closely to MBT-G principles, specifically when the therapist provides some transformative interventions, namely when she regulates the group phases and setting, when she involves the group in the mentalization of events or when she focuses on the emotional aspects of the experience. This has been the first empirical study on PM in groups. It would be important to find out if our results also hold true for other group therapies.

## Data Availability Statement

The raw data supporting the conclusions of this article will be made available by the authors, without undue reservation.

## Ethics Statement

The Ethic Committee for Clinical Experimentation of Padua -Italy (Comitato Etico per la Sperimentazione Clinica della provincia di Padova; CESC) reviewed and approved this study involving human participants (Deliberation n. 849). The patients provided their written informed consent to participate in this study.

## Author Contributions

GE, SF, and SWK designed the study and managed the literature search. GE, RP, and SWK undertook the analysis. GE, SF, RP, and SWK wrote the first draft of the manuscript and contributed to the subsequent redrafting of the manuscript. CM, VS, and SWK critically reviewed the draft of the manuscript and contributed to the interpretation of results. All authors contributed to and approved the final manuscript.

## Conflict of Interest

The authors declare that the research was conducted in the absence of any commercial or financial relationships that could be construed as a potential conflict of interest.

## Publisher’s Note

All claims expressed in this article are solely those of the authors and do not necessarily represent those of their affiliated organizations, or those of the publisher, the editors and the reviewers. Any product that may be evaluated in this article, or claim that may be made by its manufacturer, is not guaranteed or endorsed by the publisher.
